# Efficient and Accurate Zero-Day Electricity Theft Detection from Smart Meter Sensor Data Using Prototype and Ensemble Learning

**DOI:** 10.3390/s25134111

**Published:** 2025-07-01

**Authors:** Alyaman H. Massarani, Mahmoud M. Badr, Mohamed Baza, Hani Alshahrani, Ali Alshehri

**Affiliations:** 1 Computer Science and Engineering Department, The American University in Cairo, Cairo 11835, Egypt; alyamanmas@aucegypt.edu; 2 Department of Cybersecurity, College of Engineering, SUNY Polytechnic Institute, Utica, NY 13502, USA; badrm@sunypoly.edu; 3 Department of Electrical Engineering, Faculty of Engineering at Shoubra, Benha University, Cairo 11629, Egypt; 4 Department of Computer Science, College of Charleston, Charleston, SC 29424, USA; 5 Department of Computer Science, College of Computer Science and Information Systems, Najran University, Najran 61441, Saudi Arabia; hmalshahrani@nu.edu.sa; 6 Department of Computer Science, University of Tabuk, Tabuk 71491, Saudi Arabia; a.alshehri@ut.edu.sa

**Keywords:** electricity theft, anomaly detection, smart meter sensors, sensor data processing, sensor-based anomaly detection, zero-day attacks

## Abstract

Electricity theft remains a pressing challenge in modern smart grid systems, leading to significant economic losses and compromised grid stability. This paper presents a sensor-driven framework for electricity theft detection that leverages data collected from smart meter sensors, key components in smart grid monitoring infrastructure. The proposed approach combines prototype learning and meta-level ensemble learning to develop a scalable and accurate detection model, capable of identifying zero-day attacks that are not present in the training data. Smart meter data is compressed using Principal Component Analysis (PCA) and K-means clustering to extract representative consumption patterns, i.e., prototypes, achieving a 92% reduction in dataset size while preserving critical anomaly-relevant features. These prototypes are then used to train base-level one-class classifiers, specifically the One-Class Support Vector Machine (OCSVM) and the Gaussian Mixture Model (GMM). The outputs of these classifiers are normalized and fused in a meta-OCSVM layer, which learns decision boundaries in the transformed score space. Experimental results using the Irish CER Smart Metering Project (SMP) dataset show that the proposed sensor-based detection framework achieves superior performance, with an accuracy of 88.45% and a false alarm rate of just 13.85%, while reducing training time by over 75%. By efficiently processing high-frequency smart meter sensor data, this model contributes to developing real-time and energy-efficient anomaly detection systems in smart grid environments.

## 1. Introduction

The advent of electricity enabled humanity to reach heights previously unimaginable. Modern electricity grids are considered ‘smart grids’, meaning they utilize advanced infrastructure that monitors consumers’ usage and scales electricity production accordingly [1]. This is achieved by equipping consumers’ homes with smart meters (SMs), which are devices that automatically report electricity usage periodically to electric utility companies (EUCs) in near real time [2]. The electricity networks providing this key aspect of our lives, however, can sadly be prone to failure and abuse [3,4,5,6]. The reasons can roughly be classified into two categories: technical losses (TLs) and non-technical losses (NTLs). TLs refer to unavoidable energy dissipation due to equipment and infrastructure inefficiencies, such as transmission line resistance or transformer heat losses, in addition to other technical reasons. NTLs, on the other hand, occur due to human-caused factors such as electricity theft, where consumers report false electricity usage to EUCs by tampering with their SMs [7,8], financial mistakes, administrative fraud, cyber-attacks, and defective equipment, whether due to improper installation process or unauthorized modifications [9]. While TLs can be accounted for, NTLs are unpredictable and can have severe effects on people’s lives, as they can cause blackouts, brownouts [3], and huge amounts of financial losses for EUCs. Electricity theft accounts for about 10–40% of overall electricity losses around the world, totaling around USD 6 billion in the USA and GBP 175 million in the U.K. each year [10,11,12].

The literature describes two categories of methods for dealing with electricity theft: technical methods and non-technical methods. Non-technical methods include raising awareness about the effects of electricity theft, punishing it as a crime, inspecting suspects’ equipment, among others [13]. Technical methods can be further divided into three different categories: hardware-based methods, game theory-based methods, and data-based methods [14]. Hardware-based methods try to detect electricity theft through improving the hardware design of equipment to make it more resilient to tampering with and more effective at detecting any attempts at electricity theft [15]. The problem with hardware-based methods lies in their high cost and susceptibility to outside factors such as weather conditions [16]. Another potential problem with hardware-based methods is their inability to deal with new types of attacks, as electricity thieves could figure out new ways to tamper with hardware that is hard or impossible to detect, which would require constant replacements and inspections of the hardware, further increasing costs. Game theory-based methods view electricity theft as a contest between EUCs and electricity thieves, where the goal of each is to maximize their utility functions. The problem with this approach is that it is difficult and time-consuming to formulate utility functions mathematically while also keeping them accurate [17,18]. Another potential problem with this approach is the inability to function if circumstances change such that utility functions are no longer correct.

Data-based methods aim to achieve electricity theft detection using data analysis methods, most prominently using artificial intelligence (AI) and machine learning (ML). We can classify these methods into two main categories: supervised and unsupervised learning approaches. Supervised learning requires training data including electricity theft samples, while unsupervised learning trains only on normal electricity usage data [19]. Because supervised learning methods are trained on specific attack patterns, they are ineffective in detecting new attacks compared to unsupervised learning methods, which are often trained only on benign data and instead work by detecting anomalies [20]. One of the problems with all of the data-based approaches mentioned before is their high cost of running and unsustainable nature. It is estimated that generating 100 words with OpenAI’s GPT4 uses about 519 mL of water for cooling [21], and that global AI usage could be responsible for 4.2–6.6 billion cubic meters of water withdrawal in 2027. This is in addition to the fact that AI companies often underplay their role in climate change by under-representing their electricity and water usage, which can be estimated at around 762% of their reported usage [22,23]. The problem with the current generation of AI-based methods is that they often incorporate a huge dataset in the training process to produce large models, which can contribute to the AI’s role in climate change, especially when used to inspect millions of customers’ electricity usage on a regular basis.

In this paper, we uncover the potential benefits of using prototype learning to improve the size and runtime cost of the ML model. We propose prototype learning by applying both dimensionality reduction and sample reduction, representing datasets using a compact set of representative prototypes. We also combine multiple electricity theft detection models using ensemble learning techniques to overcome the shortcomings of each model and enhance the detection performance. Our electricity theft detectors are unspervised models to detect zero-day electricity theft. The Irish CER Smart Metering Project (SMP) dataset, which comprises half-hourly benign electricity consumption readings of many consumers, has been used to train the models. In our experiments, we use functions to generate malicious readings that simulate the activity of electricity thieves; this data will be used to calculate the performance metrics for our models. This paper targets electricity theft detection in environments equipped with SMs that periodically report user electricity consumption; while the method is designed with smart grids in mind, its modularity and methodology allows adaptation to other metering infrastructures with digital reporting, though it has only been tested against SMs reporting electricity usage data. Additionally, it must be noted that out of the many causes that can result in TLs and NTLs mentioned above, this paper deals specifically with the NTLs resulting from consumers tampering with their SMs to report fake data.

To the best of our knowledge, this paper is the first attempt to propose combining prototype learning and ensemble learning using one-class classifiers for zero-day electricity theft detection. The contributions of this work can be summarized as follows:

- We developed a prototype learning approach to improve the efficiency of one-class classifiers in processing electricity usage data. Specifically, we applied Principal Component Analysis (PCA) followed by K-means clustering to compress the dataset before feeding it into an ensemble-based anomaly detection pipeline for identifying novel electricity theft patterns.
- We proposed an ensemble learning framework that incorporates a meta-One-Class Support Vector Machine (OCSVM) classifier trained on the normalized output scores of two base models, the Gaussian Mixture Model (GMM) and OCSVM, each trained on prototype-reduced data, to enhance classification accuracy between benign and malicious behaviors.
- Experimental results demonstrate that the proposed method achieves a 92% reduction in dataset size and a 76.1% decrease in training time. Furthermore, it significantly improves performance compared to a standalone OCSVM model, improving accuracy from 81.52% to 88.45% and HD from 63.31% to 76.93%, while notably lowering the false alarm rate from 34.25% to 13.85%, addressing common limitations of one-class classifiers.

The paper organization is as follows. In Section 2, we discuss the related works and their limitations. In Section 3, we elaborate our methodology to integrate prototype learning with ensemble learning. In Section 4, we evaluate our methodology. Finally, we conclude the paper in Section 5.

## 2. Related Works

### 2.1. Supervised and Unsupervised Learning

The vast majority of the literature uses supervised learning techniques for electricity theft detection [24,25]. Badr et al. [20] show that this is problematic since supervised learning techniques perform badly when used for detecting zero-day attacks. The paper tests multiple supervised classification models against both attack patterns that the models were trained on and zero-day attacks that they are unaware of. Specifically, the performance of feedforward fully connected neural networks (FCNNs), convolutional neural networks (CNNs), and long-short-term memory (LSTM) supervised learning methods has been compared to that of the isolation forest (IF) and OCSVM anomaly detection algorithms. The results show that the anomaly detection methods have a clear advantage over supervised learning methods when it comes to detecting new attack patterns. Nevertheless, the highest performance metrics achieved by this paper were 83.87% for the accuracy and 84.84% for the F1 score (The F1 score can be used to evaluate detection balance, and is a balance of precision and recall. For a formal definition, see Equation (22) in Section 4.2) using the OCSVM algorithm, which are not relatively high.

Moreover, multiple other papers have found the OCSVM model to achieve good performance in the domain of electricity theft detection. Miller et al. [26] combined the OCSVM model with models based on the autoencoder architecture such that multiple OCSVM models were trained on benign data, autoencoder bottleneck outputs, and autoencoder reconstruction errors. The autoencoder was tested with two models, one implemented with the LSTM algorithm and the other is the feed-forward autoencoder (FFAE). The results showed the potential of the OCSVM to not only perform well but also combine well with other models and yielded a top performance of 79.41% accuracy and 10.22% false alarm for the OCSVM combined with the LSTM-based autoencoder model.

Another algorithm that showed great potential for combining with other models is the GMM algorithm. In [27], Liu et al. used algorithms like the GMM and K-means clustering to detect electricity theft. Before training the models, the multi-cluster feature selection (MCFS) and Principal Component Analysis (PCA) algorithms were used for feature selection, and then the detection models were trained on the feature-selected data. The results showed that the GMM outperforms the K-means algorithm, scoring better recall, precision, and F1 score.

### 2.2. Ensemble Learning

Ensemble learning has gained interest in the domain of electricity theft detection. Gunturi and Sarkar [24] designed an intelligent ML framework to identify energy fraud in smart grids based on customer energy consumption. They discussed the imbalance in data using the synthetic minority over-sampling technique (SMOTE) and evaluated different ensembles such as adaptive boost, categorical boost, extreme boost, light boost, random forests, and extra trees. Random forests and extra trees reported the highest area under the curve (AUC) at 0.90. However, their methodology was built based on supervised learning and ignored the problem of identifying a new pattern of theft. Aslam et al. [16] proposed a deep ensemble learning technique, LSTM-UNet-Adaboost, for electricity theft detection. LSTM was utilized to model temporal correlations, UNet was employed for feature extraction from two-dimensional electricity usage images, and multi-class adaptive boosting was utilized for classification. Although their method achieved high detection rates, the focus was on the known attack patterns and did not solve the problem of zero-day attack detection. In addition, the paper mentioned that they did not take into account computational cost, which is most likely high due to the increase in model numbers without proper reduction of size. The above works’ limitations are their use of supervised learning and labeled datasets; while they excel in detecting known patterns of electricity, they may fail to detect emerging ones.

### 2.3. Prototype Learning

Prototype learning is a collection of techniques whereby the size of a model is reduced by training on the prototypes of the dataset instead of the whole dataset. Recently, prototype learning has emerged as a promising approach to classification problems, including anomaly detection, due to its ability to reduce data sizes and still improve results at times. Viegas et al. [28] applied prototype learning to help in detecting NTLs in smart grids. They first extracted prototypes from SM data with Gustafson–Kessel (GK) fuzzy clustering, which became representative of typical consumption behaviors. New data samples were then classified as anomalous if their distance to the prototypes exceeded a threshold. Although the authors considered zero-day attack detection, the detection performance was relatively low. Moreover, Sun et al. [19] combined prototype learning with CNN and LSTM to detect electricity theft. They employed CNN and LSTM to extract features from electricity consumption data and utilized prototype learning to derive representative prototypes from each class. After constructing the prototypes of normal and abnormal consumption patterns, new samples are classified by their similarities to the constructed prototypes. Their proposed method showed good performance for imbalanced datasets. However, this method was based on supervised learning with labeled training data and therefore, cannot detect new attack patterns. Furthermore, it fails in optimizing computational efficiency.

### 2.4. Limitations of Existing Techniques

Despite significant advancements in electricity theft detection methods, the existing literature suffers from some major limitations that our approach aims to enhance. One of the main limitations in many studies is the trade-off between detection capability and computational efficiency. For supervised learning approaches, such as those presented in [16,19,24], the models are accurate in detecting known attack patterns but are restricted in their capability to identify zero-day attacks. In other words, the models are restricted to identifying theft patterns that have been encountered in training and consequently leave the EUCs vulnerable to emerging attack vectors. Unsupervised techniques, such as those proposed in [20,26,27,28], are capable of detecting zero-day attacks; however, they often face significant efficiency limitations. Badr et al. [20] reported modest accuracy (83.87%) using OCSVM, but their method lacked data reduction strategies, limiting its scalability in large-scale environments. Miller et al. [26] combined OCSVM with autoencoders to enhance detection, but this came at a high computational cost, without addressing the need for data compression. Liu et al. [27] employed GMM with feature selection, yet implemented only limited data reduction and did not significantly reduce computational complexity. Viegas et al. [28] used GK clustering for zero-day attack detection but reported lower performance (AUC: 0.741) and did not incorporate prototype learning to improve computational efficiency. These approaches collectively highlight the challenge of balancing detection performance with computational scalability in unsupervised settings.

Table 1 compares our proposed method to the existing methods. A common drawback of the existing works is their high resource consumption during the training and inference phases. Most existing approaches must process the entire dataset without effective data compression techniques, and real-time detection is challenging in large-scale smart grid deployments with millions of customers and high-frequency measurements. The aforementioned limitations highlight the primary research gap to develop a method that can simultaneously achieve high detection accuracy for zero-day attacks while significantly reducing computational requirements through efficient data compression. Our proposed methodology directly bridges this gap by integrating prototype learning with meta-level ensemble classification to retain detection functionality while dramatically reducing dataset size and processing requirements.

## 3. Methodology

This section details our methodology for designing an efficiently trained electricity theft detector capable of accurately detecting zero-day electricity theft cases. First, we develop a prototype learning approach to reduce the training dataset into compact representative prototypes. Then, we train one-class classifiers on the extracted prototypes to detect zero-day attacks. Finally, we propose an ensemble learning framework incorporating multiple classifiers to enhance the detection performance.

### 3.1. Efficient Training Through Prototype Learning

In this paper, we propose a comprehensive prototype learning approach for compressing the data horizontally and vertically through PCA [29] and K-means clustering algorithm [28], respectively. Specifically, the PCA is used to reduce the dimensionality of the electricity usage data and the K-means clustering algorithm is used to derive prototypes from the electricity usage data.

PCA is a dimensionality reduction technique that transforms high-dimensional data into a low-dimensional space in such a way that it retains as much of the data’s variance as possible. It achieves this by applying the following steps given in Algorithm 1.

*k* can be selected to be the number of components that explains a specified percentage of the variance of the data. Given a value smaller than the orignal number of features, this greatly cuts down the computational cost in subsequent steps while retaining the key patterns in the data which will be used for ETD.
**Algorithm 1** PCA.**Input:** X_input, *k* **Output:** X_transformed  1:X_std ← standardize(X_input)  2:cov_matrix ← (X_std^T^ · X_std)/(*n* − 1)  3:evectors, evalues ← eigendecomposition(cov_matrix)  4:Rearrange each pair of evectors and evalues in descending order of evalues  5:W ← evectors_1:*k*,·_  6:X_transformed ← X_std · W  7:**return** X_transformed

Algorithm 1 presents the step-by-step process of Principal Component Analysis. The algorithm takes the input dataset X_input (an n×d matrix where *n* is the number of samples and *d* is the number of features) and the desired number of principal components *k* (where k<d). The algorithm returns X_transformed, the dataset with reduced dimensionality.

The process begins with standardizing the input data (X_std) such that all features have zero mean and unit variance, which is necessary for PCA since features with larger scales would otherwise overpower the analysis. Then, the covariance matrix is estimated as (X_std^T^ · X_std)/(*n* − 1), where X_std^T^ is the transpose of the standardized data matrix, and (n−1) provides the unbiased estimator of covariance.

The eigendecomposition procedure computes the eigenvectors (evectors) and eigenvalues (evalues) of the covariance matrix. The eigenvectors provide the principal components (directions of maximal variance), and the eigenvalues provide the variance accounted for by each component. These eigenvector–eigenvalue pairs are arranged in decreasing order of eigenvalues so that components accounting for the most variance are prioritized.

The transformation matrix W is obtained by selecting the first *k* eigenvectors (${\mathrm{evectors}}_{1:k,\cdot }$, i.e., rows 1 to *k* and all columns). Then, reduced dimensional data X_transformed is obtained by projecting the standardized data onto the selected principal components: X_transformed = X_std · W.

K-means clustering algorithm reduces the number of samples in our dataset by grouping similar patterns of electricity consumption into K clusters and representing each cluster with its centroid. The algorithm has the following steps:

1. Initialize K centroids randomly in the feature space.
2. Repeat until convergence:
  - Assign each sample to the nearest centroid depending on the Euclidean distance.
  - Update each cluster’s centroid to be the mean of all samples in the cluster.

In our work, we apply the K-means algorithm on the PCA-reduced data to cluster the training samples according to similar consumption patterns. The centroids of these clusters are prototypes of typical electricity consumption. This drastically reduces the memory and computational cost of training and running our electricity theft detection models. Employing the K-means algorithm for prototype selection brings the following benefits:

- It naturally finds representatives of different consumption patterns.
- The centroids average out random variations while preserving systematic patterns.
- The number of prototypes can be specified.
- It complements the PCA since it reduces samples instead of features.
- The centroids can be directly used to efficiently train the one-class classifiers.

Together, the PCA and the K-means algorithm form an effective two-step data reduction process that compresses both the feature dimension and the number of samples while retaining the key features that enable electricity theft detection. Our data reduction approach, combing the PCA and the K-means algorithms, is illustrated in Algorithm 2.

Algorithm 2 encapsulates our overall data compression approach that combines PCA and K-means clustering to compress electricity usage data in both the horizontal and vertical dimensions. The algorithm takes the original training set training_X (an n×d matrix, where *n* is the number of training instances and *d* is the number of features), the test set testing_X (an m×d matrix), the test labels testing_y, the number of principal components to keep number_components (*k*), and the number of clusters number_clusters (*K*). The algorithm returns training_X_centroids (the *K* cluster centers for prototype consumption profiles) and testing_X_pca (PCA-transformed test data).
**Algorithm 2** Our proposed data-size reduction approach.**Input:** training_X, testing_X, testing_y, number_components, number_clusters **Output:** training_X_centroids, testing_X_pca
  1:pca_var ← PCA(number_components)  2:pca_var.fit(training_X)  3:training_X_pca ← pca_var.transform(training_X)  4:testing_X_pca ← pca_var.transform(testing_X)  5:kmeans_var ← KMeans(number_clusters)  6:kmeans_var.fit(training_X_pca)  7:training_X_centroids ← kmeans_var.cluster_centers  8:**return** training_X_centroids, testing_X_pca

The algorithm begins by creating a PCA instance with the provided number of components *k* and fitting it onto training data to learn principal components accounting for maximum variance in electricity use patterns. The fitted PCA transformation is then used on the training data and test data, reducing their dimensions from *d* to *k* features without compromising the underlying consumption attributes. This horizontal dimension reduction significantly reduces computational complexity for subsequent processing operations.

Following dimension reduction, a K-means clustering algorithm is initialized with *K* clusters and trained on the PCA-transformed training data training_X_pca. The K-means algorithm groups into sets of similar patterns of electricity usage and computes the centroid of each set, the average usage pattern for each group. These cluster centroids are prototypes that select the most significant features of different usage behaviors and shrink the size of the dataset from *n* training examples to *K* representative prototypes. The algorithm provides such centroids as the compressed training set such that they may be utilized for training electricity theft detection models effectively at minimal loss of discriminative information.

Please note that in cases with very highly variable data, the proposed combined PCA + K-means prototype learning approach might struggle to capture the patterns in the data with poorly chosen parameters for the PCA and K-means algorithm. For this reason, it becomes important to fine-tune the models and test them to ensure their reliability. A method for doing so is explained in Section 4.

### 3.2. Zero-Day Detection Through One-Class Classification

To detect zero-day electricity theft, two one-class classifiers are employed, OCSVM and GMM. These classifiers are trained only on one class of data, and then detect whether a given sample belongs to that class. This makes them well-suited to our case. Furthermore, selecting one-class classifiers for this task allows us to deal with the problem of imbalanced datasets, which is a common problem for anomaly detection. Imbalanced datasets are datasets where one class of data is more dominant in its presence relative to other classes of data. When dealing with ETD datasets, handling imbalance in the data is essential since most consumers are not stealing electricity, while the thieves are the minority. This makes such datasets inherently imbalanced. This is a problem when trying to train a model on multiple classes of data, since most algorithms assume that classes are roughly equivalently present in the data, and struggle to train against small amounts of malicious data. However, since in the case of this methodology, only one-class classifiers are used, the class imbalance is not a problem since such models only train on the majority class, which is well represented.

OCSVM is selected based on its proven effectiveness in electricity theft detection, as demonstrated in prior studies [20]. GMM is chosen for its probabilistic modeling capability, which allows it to estimate the underlying data distribution and assign likelihoods to new observations, making it a strong candidate for anomaly detection. The remainder of this subsection describes how the two algorithms work.

#### 3.2.1. One-Class Support Vector Machine (OCSVM)

Like other support vector machine algorithms, OCSVM is a classification algorithm. However, what makes it different is that it is a one-class classifier. This means that it only trains on one class of data and tries to detect afterwards if a given input belongs to that class or not. It does so by learning a small region that contains most of the training points in a high-dimensional feature space. This is achieved by finding a maximally separating hyperplane between the training data and the origin in that feature space. If we have training points $X_{j}$, mapped into a feature space using a kernel function k(·,·), OCSVM solves the following optimization problem:

(1) $$\begin{array}{c} \min_{w,\rho ,\xi }\frac{1}{2}{∥w∥}^{2}+\frac{1}{\nu \ell }\sum_{j}\xi_{j}-\rho \end{array}$$

subject to

(2) $$\begin{array}{c} \left(w\;\cdot \;\Phi \left(X_{j}\right)\right)\geq \rho -\xi_{j},\;\xi_{j}\geq 0 \end{array}$$

where *w* and ρ define the hyperplane, $\xi_{j}$ are slack variables, Φ(·) is the feature mapping function that maps input data to a high-dimensional feature space (induced by the kernel function k(·,·)), *ℓ* is the number of training points, and ν∈[0,1] is a parameter that controls the percentage of training points that are allowed to fall outside the learned region. Then, the decision function for a new point *X* can be defined as such:

(3) $$\begin{array}{c} f(X)=\mathrm{sgn}((w\;\cdot \;\Phi (X))-\rho ) \end{array}$$

The return of this function is +1 for points that are inside the learned region and −1 for points considered as outliers [30]. However, it returns only a binary output, which will not be sufficient for our purposes, since we need to combine multiple models using ensemble learning techniques, as outlined in Section 3.3. For this purpose, we can use the raw decision value without the sgn function to get a continuous score as follows:

(4) $$\begin{array}{c} f_{score}\left(X\right)=\left(w\;\cdot \;\Phi \left(X\right)\right)-\rho \end{array}$$

The output of the above equation represents the signed distance from *X* to the decision boundary in feature space. The more negative it is, the more likely *X* is an outlier.

#### 3.2.2. Gaussian Mixture Model (GMM)

This algorithm works by first assuming the data to be generated by a model M, which is a mixture of *k* multivariate Gaussian/normal distributions with *d* variables, and then trying to fit the data to said distributions. If we represent the *r*th Gaussian mixture component by the following equation:

(5) $$\begin{array}{c} f^{r,\Theta }\left(\bar{X_{j}}\right)=\frac{\exp\left[-\frac{1}{2}\left(\bar{X_{j}}-\bar{\mu_{r}}\right)\Sigma_{r}^{-1}{\left(\bar{X_{j}}-\bar{\mu_{r}}\right)}^{T}\right]}{\sqrt{|\Sigma_{r}|}\;\cdot \;{\left(2\pi \right)}^{\frac{d}{2}}} \end{array}$$

where $\bar{\mu_{r}}$ is the mean of the component in *d* dimensions, and $\Sigma_{r}$ is the covariance matrix of the component with size d×d. Let $\alpha_{r}$ intuitively represent the proportion of samples of the model M that were generated by component *r*, then we can use the density function shown in Equation (6) to calculate a value that represents how likely it is for a data point $\bar{X_{j}}$ to have been generated by model M. This can be used as an outlier score for point $\bar{X_{j}}$, which can later be used to calculate the probability that the point is an outlier [31].

(6) $$\begin{array}{c} f^{point}\left(\bar{X_{j}}\right)=\sum_{i=1}^{k}\alpha_{i}\;\cdot \;f^{i}\left(\bar{X_{j}}\right) \end{array}$$

### 3.3. Accurate Detection Through Ensemble Learning

Ensemble learning is an ML paradigm that seeks to combine multiple models into a system that is robust and accurate, much more so than any of the individual models. Two main approaches to ensemble learning are bagging and boosting. Bagging (Bootstrap Aggregating) means training several instances of the same ML algorithm on different random subsets of the training dataset. Random forest is a popular example of Bagging. Boosting, on the other hand, trains ML models sequentially, where each next model tries to correct the errors of the previous models. AdaBoost and XGBoost are two common examples of Boosting. AdaBoost adjusts the weights of training samples to give more importance to misclassified examples in the next iteration; XGBoost is a boosting technique where regularization and tree pruning are incorporated to improve speed and performance. In our work, we implement a third ensemble learning technique, called stacking, where we combine multiple different types of one-class classifiers that each capture different aspects of normal electricity consumption patterns. Specifically, we propose a meta-OCSVM ensemble framework that learns to combine the predictions of multiple one-class classifiers. Rather than simple score averaging, this approach treats the base model outputs as features for a higher-level classifier that can learn complex decision boundaries.

#### 3.3.1. Score Space Transformation

The meta-OCSVM operates on a transformed feature space consisting of the decision scores from both OCSVM and GMM. Given a sample *o*, we obtain

(7) $$\begin{array}{c} s_{ocsvm}\left(o\right)=\left(w\;\cdot \;\Phi \left(X\right)\right)-\rho \end{array}$$

(8) $$\begin{array}{c} s_{gmm}\left(o\right)=f^{point}\left(o\right)=\sum_{i=1}^{k}\alpha_{i}\;\cdot \;f^{i}\left(o\right) \end{array}$$

where $s_{ocsvm}\left(o\right)$ represents the signed distance to the OCSVM decision boundary and $s_{gmm}\left(o\right)$ is the density estimation from the GMM model.

#### 3.3.2. Score Normalization

Since OCSVM and GMM produce scores on different scales and with different distributions, we normalize them using the MinMaxScaler before passing them to the meta-classifier as follows:

(9) $$\begin{array}{c} s_{norm}\left(o\right)=\frac{s\left(o\right)-s_{min}}{s_{max}-s_{min}} \end{array}$$

This transformation brings all scores into the [0,1] range while preserving their relative ordering.

#### 3.3.3. Meta Classification

The normalized scores form a 2-dimensional feature space $X_{meta}=\left[s_{norm\_ocsvm},s_{norm\_gmm}\right]$ on which we train a meta-classifier, another OCSVM, as follows:

(10) $$\begin{array}{c} \min_{w,\rho ,\xi }\frac{1}{2}{∥w∥}^{2}+\frac{1}{\nu \ell }\sum_{j}\xi_{j}-\rho \end{array}$$

subject to

(11) $$\begin{array}{c} \left(w\;\cdot \;\Phi \left(X_{meta_{j}}\right)\right)\geq \rho -\xi_{j},\;\xi_{j}\geq 0 \end{array}$$

where ν controls the fraction of training points allowed to be outliers and *ℓ* is the number of training points. This meta-classifier learns to identify regions in score space that correspond to normal behavior across both base models. The meta-OCSVM uses an RBF kernel with gamma=‘scale’ and ν=0.115, allowing it to learn non-linear decision boundaries in the score space. This configuration enables the model to capture complex relationships between the base classifier predictions while maintaining robust generalization.

#### 3.3.4. Final Prediction

**Figure 1:**
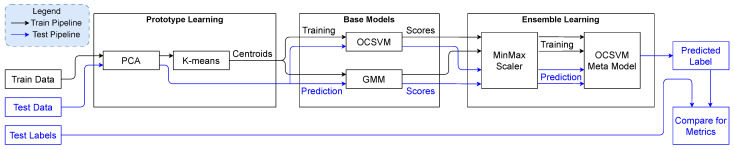
The entire framework to train the meta-OCSVM and use it to make predictions, from processing the training data to predicting labels for the testing data.

For a new sample *o*, the final prediction is determined by

(12) $$\begin{array}{c} f_{meta}\left(o\right)=\mathrm{sgn}\left(\left(w\;\cdot \;\Phi \left(\left[s_{norm\_ocsvm}\left(o\right),s_{norm\_gmm}\left(o\right)\right]\right)\right)-\rho \right) \end{array}$$

where +1 indicates an inlier (normal behavior) and −1 indicates an outlier (electricity theft). The entire framework to train the meta-OCSM and use it to make predictions is shown in Figure 1.

## 4. Performance Evaluation

### 4.1. Dataset and Attack Model

The Irish Commission for Energy Regulation (CER) Smart Metering Project (SMP) dataset [32] has been used to train and test our electricity theft detection models. The dataset spans across 3600 residential units, having half-hourly electricity consumption readings per unit for 536 days, providing around 25,728 readings per unit. For our experiments, a random subset of 40 units was chosen from the dataset. Since we are interested in daily electricity theft detection, we reshape the data of each unit to samples of 48 half-hour readings each, such that each unit provides 536 samples, for a total of 21,440 samples.

Since the data provided by the SMP dataset is all benign, malicious samples are created using the attack model described below that imitates the electricity theft behavior. The attack model used in this paper is similar to that in [20], which includes the most common attack functions found in the literature, described by the equations below. Please note that due to our using a one-class classifier for anomaly detection, our proposed methods should be able to detect any type of attack on smart meters that makes use of reporting manipulated data to the EUC, resulting in deviations from normal electricity usage patterns. The proposed methodology, therefore, should be able to deal with more than the below 6 attack types, and the attack types used for testing are used to test the effectiveness of our detector against attacks in general, and should give an idea of how well it can handle other attacks as well.

(13) $$R_{1c}\left(d,t\right)=r_{1}M_{c}\left(d,t\right)$$

(14) $$R_{2c}\left(d,t\right)=r_{2}\left(d,t\right)M_{c}\left(d,t\right)$$

(15) $$\begin{array}{cc} R_{3c}\left(d,t\right) & =\left\{\begin{array}{cc} 0 & \forall t\in [t_{s}\left(d\right),t_{e}\left(d\right)]\\ M_{c}\left(d,t\right) & \forall t\notin [t_{s}\left(d\right),t_{e}\left(d\right)] \end{array}\right. \end{array}$$

(16) $$R_{4c}\left(d,t\right)=\mathrm{mean}(M_{c}\left(d\right))$$

(17) $$R_{5c}\left(d,t\right)=r_{3}\left(d,t\right)\mathrm{mean}\left(M_{c}\left(d\right)\right)$$

(18) $$R_{6c}\left(d,t\right)=M_{c}\left(d,T-t+1\right)$$

where $M_{c}\left(d,t\right)$ is the real electricity usage for customer *c* on day *d* for time interval *t*, while $R_{ic}\left(d,t\right)$ is the electricity usage of said customer after the attack function is applied.

Equation (13) describes an attack where electricity thieves simply multiply their electricity usage by a positive constant factor $r_{1}$ that is less than 1. Similarly, Equation (14) describes an attack whereby thieves multiply their electricity usage by a number, but in this case, the number is a function of the date and time. This is carried out by thieves to report lower electricity usage while electricity prices are high. In Equation (15), the attacker either reports normal electricity usage or zero electricity usage depending on the date and time. Similar to Equation (14), this can be conducted to reduce reported usage during times when electricity is expensive. Equation (16) describes an attack where the thief constantly reports the average usage across an entire day. Equation (17) tries to chain techniques from both attacks (14) and (16) by taking the mean usage of electricity in a day, and then multiplying it by a factor that is a function of date and time. Finally, Equation (18) describes an attack whereby the electricity thief reports electricity usage from a different time to account for the fact that electricity prices change to minimize the electricity bill.

### 4.2. Metrics

To fine-tune the parameters of our electricity theft detection models and evaluate their performance, we use a group of classification metrics. These metrics are derived from the following four numbers:

- **TP (True Positives):** the number of malicious samples correctly identified.
- **FP (False Positives):** the number of benign samples incorrectly identified as malicious.
- **TN (True Negatives):** the number of benign samples correctly identified.
- **FN (False Negatives):** the number of malicious samples incorrectly identified as benign.

In this paper, we use the following evaluation metrics:

1. **Accuracy:** The percentage of samples identified correctly as what they are. It can be calculated as follows:
  (19) $$\begin{array}{c} \mathrm{ACC}\%=\frac{\mathrm{TP}+\mathrm{TN}}{\mathrm{TP}+\mathrm{TN}+\mathrm{FP}+\mathrm{FN}}\times 100 \end{array}$$
2. **Confusion Matrix:** This is a 2×2 matrix that has the TN, FN, TP, and FP at indices (0,0), (1,0), (1,1), and (0,1), respectively.
3. **Recall:** Measures the ratio of malicious samples identified as malicious.
  (20) $$\begin{array}{c} \mathrm{RE}\%=\frac{\mathrm{TP}}{\mathrm{TP}+\mathrm{FN}}\times 100 \end{array}$$
4. **Precision:** Measures the proportion of correctly identified malicious samples among all samples labeled as malicious by the model.
  (21) $$\begin{array}{c} \mathrm{PR}\%=\frac{\mathrm{TP}}{\mathrm{TP}+\mathrm{FP}}\times 100 \end{array}$$
5. **F1 Score:** As shown by the formula below, it represents a balance between the recall and precision.
  (22) $$\begin{array}{c} F1\%=\frac{2\times \mathrm{TP}}{2\times \mathrm{TP}+\mathrm{FP}+\mathrm{FN}}\times 100 \end{array}$$
6. **False Alarm Rate (FAR):** The proportion of negative points that were incorrectly classified as positive. It can be calculated according to the following formula:
  (23) $$\begin{array}{c} \mathrm{FAR}\%=\frac{\mathrm{FP}}{\mathrm{FP}+\mathrm{TN}}\times 100 \end{array}$$
7. **Highest Difference (HD):** Shows how well a classifier can distinguish between malicious and benign samples. It is calculated as follows:
  (24) $$\begin{array}{c} \mathrm{HD}\%=\mathrm{RE}\%-\mathrm{FAR}\% \end{array}$$
8. **Area Under the Receiver Operating Characteristic Curve (AUC):** The Receiver Operating Characteristic Curve (ROC) represents the relationship between TP and FP as the threshold for classification changes. The AUC represents a model’s ability to distinguish between different classes.
9. **Dataset Size Reduction:** To judge the effectiveness of our prototype learning approach in reducing the size of the dataset, Equation (25) shows the overall percentage of data points/components of the reduced dataset relative to the original dataset as a function of the number of clusters $n_{c}$, number of original data points $n_{o}$, number of components after PCA $c_{p}$, and original number of components $c_{o}$.
  (25) $$\begin{array}{c} s\left(n_{c},n_{o},c_{p},c_{o}\right)\%:=\frac{c_{p}n_{c}}{c_{o}n_{o}}\times 100 \end{array}$$

The accuracy, confusion matrix, recall, precision, F1 score, false alarm rate, highest difference, and AUC are used to determine the effectiveness of our ML models in detecting electricity theft while not misidentifying benign data as malicious. Meanwhile, the dataset size reduction and the time taken to train the ML model are used to judge the effectiveness of our prototype learning approach in achieving efficiency.

### 4.3. Experiments

Two experiments were conducted to evaluate the effectiveness of our proposed methodology. The first experiment assesses how the prototype learning approach reduces the dataset size while preserving the ability to train high-performing models. The second experiment involves training multiple ML models on the prototype data and combining them using the proposed ensemble learning framework. All of the experiments were run on a Dell G15 5520 device with the specifications shown in Table 2.

#### 4.3.1. Experiment 1

**Figure 2:**
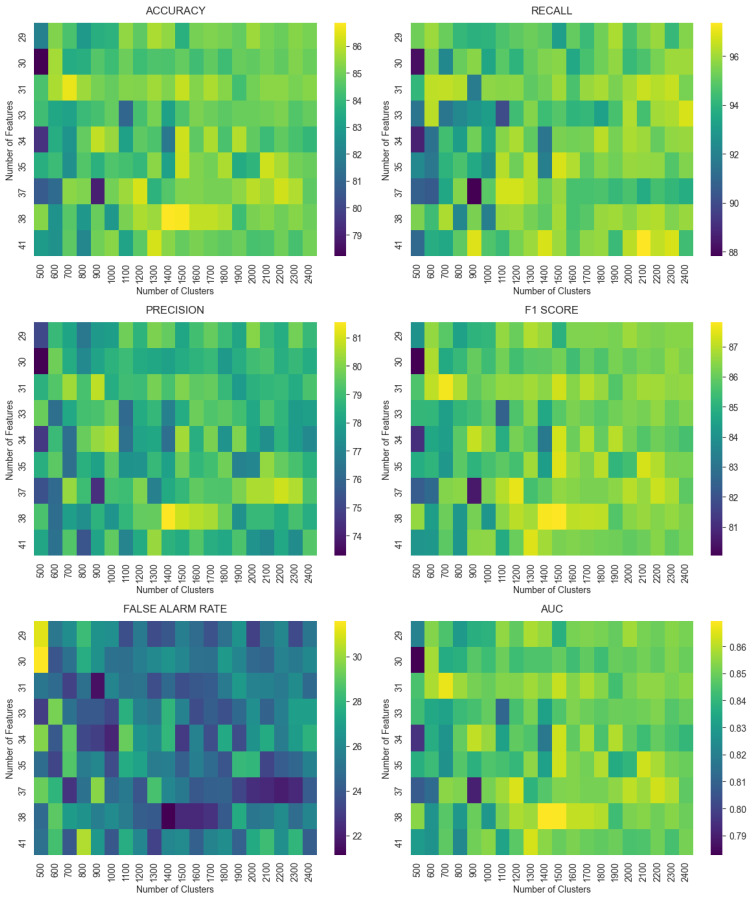
Performance metrics visualized against the number of clusters and the number of features, representing the trade-off between efficiency and detection performance.

**Figure 3:**
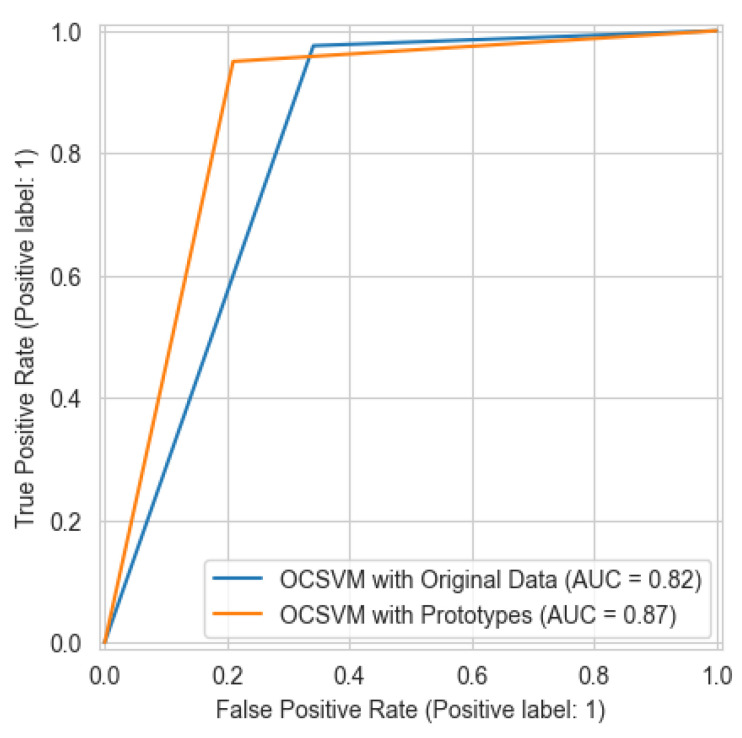
The ROC curve for the OCSVM before and after prototype learning.

In this experiment, we aim to train a one-class classifier on a reduced-size dataset while still achieving good detection performance. We chose to train an OCSVM, as it demonstrated strong detection performance in earlier studies [20]. Figure 2 visualizes the performance metrics for an OCSVM trained on the data produced through our prototype learning approach, with the number of clusters for the K-means algorithm on the x-axis and the percentage of features for the PCA on the y-axis. From the figure, we can observe that we consistently have the best metrics when the number of clusters is 1400 and the number of features is 38; we therefore choose these values for our parameters. This observation can be more systematically automated by using the Grid Search algorithm shown in Algorithm 3 with the number of clusters for K-means and the number of features for PCA as the variables to optimize and a metric like F1 or an aggregate metric of multiple other ones as the scoring function to maximize. Using Equation (25), we can see that the chosen values for our parameters for PCA and K-means produce a dataset that is approximately 7.7% the size of the original dataset, while also producing an OCSVM model that achieves a strong detection performance, as seen in Table 3 and Figure 3.
**Algorithm 3** The Grid Search algorithm for fine-tuning the models.**Input:** Vector of *n* sets of parameters *P*, for a specific classification algorithm *a*. Scoring function *s* that is to be maximized. **Output:** Set of parameters *p* that produces the highest value for *s*  1:param_combinations ← CartesianProduct(*P*)  2:maximal_score ← −∞  3:best_params ← None  4:**for** *p* ∈ param_combinations **do**  5:  model←a(p)  6:  score←s(model)  7:  **if**  score > maximal_score **then**  8:    maximal_score ← score  9:    maximal_params ← *p* 10:  **end if** 11:**end for** 12:**return** best_params

While it might be expected that prototype learning achieves efficiency at the cost of reduced detection performance metrics, given the fact that prototype learning provides reduced number of samples to train the models, the opposite was interestingly observed. The performance of the OCSVM generally improved after fitting it on prototype data, as seen in Table 3 and Figure 3. We can attribute this improvement to several factors. First, the K-means clustering creates more meaningful representations in the feature space by focusing on the most representative consumption patterns while reducing the influence of anomalous data points. Second, as demonstrated by Sun et al. [19], prototype learning pays more attention to the overall similarity of features rather than partial information, creating clearer decision boundaries for classification. In addition, the prototype learning approach proposed in this paper likely enhanced the feature learning process by filtering out noise and focusing on core consumption patterns, which would allow the OCSVM classifier to establish more robust decision boundary despite using significantly less data. This finding aligns with research showing that prototype learning can improve model robustness while simultaneously reducing computational requirements [19].

#### 4.3.2. Experiment 2

In this experiment, we evaluate the performance of our proposed ensemble learning framework compared to that of the base models. For obtaining the best results, we employed the Grid Search optimization algorithm, described in Algorithm 3, to fine-tune the hyperparameters of the models. The optimal hyperparameters of the GMM model is given in Table 4. The F1 score was selected as the optimization metric because it balances precision and recall, which is particularly important given the typically high false positive rates associated with one-class classification tasks. For the OCSVM classifiers, the critical parameter ν controls the fraction of training points allowed to be outliers, directly impacting the model’s decision boundary. We optimized this parameter using the SciPy minimize function with the following objective:

(26) $$\begin{array}{c} \nu^{*}=\underset{\nu }{arg min}\frac{1}{\mathrm{accuracy}(\nu )} \end{array}$$

subject to 0<ν≤1. The optimization process employed the Nelder–Mead simplex algorithm, yielding the optimal hyperparameters given in Table 5.

**Figure 4:**
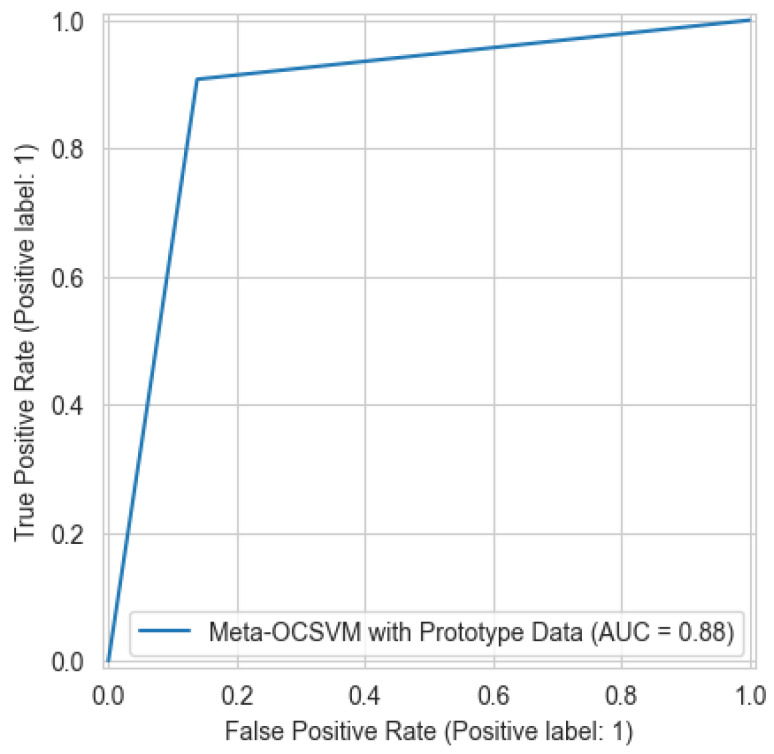
The ROC curve for the Meta-OCSVM with prototype learning.

**Figure 5:**
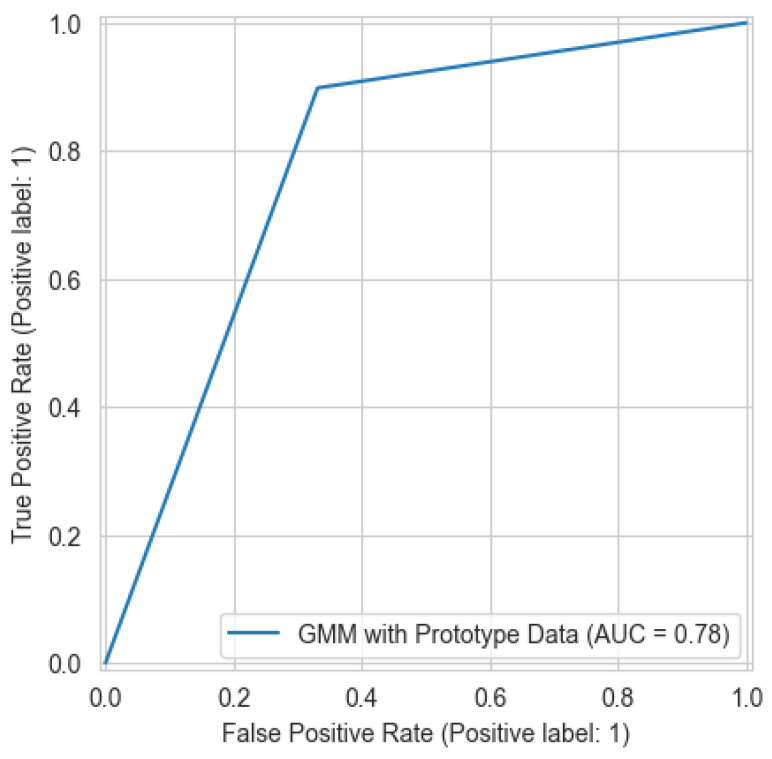
The ROC curve for the GMM base classifier with prototype learning.

The meta-OCSVM model demonstrates significant improvements in overall detection performance over both of the base models. As shown in Table 3, the meta-OCSVM achieves the highest accuracy (88.45%) among all models while substantially reducing the false alarm rate to 13.85%. Moreover, comparing Figure 4 to Figure 3 and Figure 5, it is clearly observed that the meta-OCSVM achieves the highest AUC (88.46%). This marked improvement can be attributed to the meta-classifier’s ability to learn optimal decision boundaries in the normalized score space, effectively leveraging complementary information from both base models. In terms of computational efficiency, our approach maintains a clear advantage in reducing training time. Table 6 compares the total training time of the electricity theft detection model with and without prototype learning. By reducing the dataset to approximately 8% of its original size, our method achieves a 76.1% reduction in training time.

## 5. Conclusions and Future Work

In this work, we presented a hybrid model that integrates prototype learning with a meta-level ensemble learning technique to address the challenges of electricity theft detection in smart grids. Our approach condensed the dataset using a combination of PCA and K-means clustering, effectively preserving core consumption patterns essential for anomaly detection while significantly reducing computational overhead. This enabled the efficient training of our base one-class classifiers, OCSVM and GMM, whose predictions were then combined through a meta-OCSVM operating in a normalized score space. The ensemble framework demonstrated notable effectiveness in learning an optimal decision boundary by leveraging the complementary strengths of the base classifiers. By treating normalized anomaly scores as features, our approach achieved superior detection accuracy (88.45%) of zero-day electricity theft and substantially reduced the false alarm rate to 13.85%, outperforming individual models. Our experimental results on a real-world dataset provide strong evidence of the proposed method’s efficacy. Reducing the training dataset to just 8% of its original size led to a 76.1% decrease in training time, without sacrificing detection performance. These results underscore the potential of the proposed model for scalable deployment in smart grid environments. Furthermore, the meta-learning framework’s ability to capture non-linear relationships among base classifier outputs proved valuable for enhancing detection robustness.

We find that the proposed method requires no further modification at this time as it already achieves both high zero-day detection accuracy and significant reduction in computational requirements. The efficiency and robustness of the two-step prototype learning and meta-ensemble pipeline are verified through our empirical results and are straightforward to extend to other one-class classifiers or meta-model architectures without alteration of the underlying pipeline.

Future research could explore several promising avenues. First, investigating alternative architectures for the meta-classifier, such as deep neural networks or Gaussian processes, may help capture more complex relationships in the score space. Additionally, the framework could be extended to incorporate a wider variety of base models, including those based on deep learning. Furthermore, future work may explore integrating efficiency-focused methods beyond prototype learning, such as employing the Stochastic Gradient Descent One-Class Support Vector Machine (SGDOCSVM), which offers linear time complexity and may further reduce training time without compromising detection accuracy. Finally, due to the lack of real-world testing in this paper, future research should confirm that this method works in the real world by collaborating with electricity utility companies to deploy the model to the real world and see how well it performs, noting any potential shortcomings and addressing them.

## Figures and Tables

**Table 1:** Comparative analysis of state-of-the-art methods and our proposed approach.

Study	Detection Approach	Zero-Day Attacks Detection	Improved Performance through Ensemble Learning	Efficiency Through Prototype Learning	Achieving Efficiency While Keeping High Performance
Badr et al. [20]	Unsupervised (OCSVM, IF)	✓	×	×	×
Miller et al. [26]	Unsupervised (OCSVM + Autoencoder)	✓	✓	×	×
Liu et al. [27]	Unsupervised (GMM + MCFS)	✓	×	×	×
Gunturi & Sarkar [24]	Supervised (AdaBoost, XGBoost…)	×	✓	×	×
Aslam et al. [16]	Supervised (LSTM-UNet-Adaboost)	×	✓	×	×
Sun et al. [19]	Supervised (CNN-LSTM)	×	✓	×	×
Viegas et al. [28]	Unsupervised (GK Clustering)	✓	×	×	×
Proposed Method	Unsupervised (PCA+K-means + Meta-OCSVM)	✓	✓	✓	✓

• In this table, the check mark (✓) means that the feature was implemented, while the cross (×) means that it was not. So for example for the column labeled ‘Zero-Day Attacks Detection,’ if a row has for that column a check mark, it means the paper’s proposed approach is able to detect zero-day attacks, while a cross would imply that the paper’s approach is unable to detect zero-day attacks. • Sun et al. [19] does use prototype learning; however, not for dataset size reduction, but rather for achieving better classification metrics.

**Table 2:** Dell G15 5520 specifications.

Component	Specification
Processor	Intel Core i7-12700H
Number of Cores	20 cores
Processor Speed	Up to 4.7 GHz
RAM	16 GB
RAM Type	DDR5
Graphics Card	NVIDIA GeForce RTX 3060 Mobile / MaxQ
Graphics Memory	6 GiB
Operating System	NixOS 24.11
Python	CPython 3.11
NumPy	v1.26.4
Scikit-learn	v1.5.2

**Table 3:** Comparison of the electricity theft detection models.

Model	Acc	RE	PR	F1	FAR	HD	AUC
OCSVM before prototype learning	81.52	97.56	73.69	83.96	34.25	63.31	0.8165
OCSVM after prototype learning	86.86	94.99	81.55	87.76	21.13	73.86	0.8693
GMM after prototype learning	78.47	89.82	72.99	80.53	32.68	57.14	0.7857
**Meta-OCSVM after prototype learning (Proposed)**	**88.45**	90.78	86.57	**88.62**	13.85	**76.93**	**0.8846**

**Table 4:** The optimal hyperparameters of the GMM model.

Hyperparameter	Value
Number of Components (n_components)	208
Tolerance (tol)	0.0001
Covariance Type	spherical
Contamination	0.1
Random State	42

**Table 5:** The optimal hyperparameters of the OCSVM models.

Hyperparameter	Base OCSVM	Meta OCSVM
Kernel	sigmoid	rbf
Gamma	scale	scale
Nu (ν)	0.02	0.115

**Table 6:** Time spent on training models with and without prototype learning. Note that each time, measurement was performed 5 times and then the average was taken.

	Model	Training Time
Without Prototypes	OCSVM	0.311 s
	GMM	13.073 s
	Meta-OCSVM	1.342 s
	Total	14.726 s
With Prototypes (Proposed)	PCA	0.006 s
	K-means	1.621 s
	OCSVM	0.036 s
	GMM	1.691 s
	Meta-OCSVM	0.179 s
	Total	3.533 s

## Data Availability

The original contributions presented in the study are included in the article [32].
